# Targeting HDACs: A Promising Therapy for Alzheimer's Disease

**DOI:** 10.1155/2011/143269

**Published:** 2011-09-20

**Authors:** Ke Xu, Xue-Ling Dai, Han-Chang Huang, Zhao-Feng Jiang

**Affiliations:** ^1^College of Life Science, Capital Normal University, Beijing 100048, China; ^2^College of Arts and Science, Beijing Union University, Beijing 100191, China; ^3^Department of Physiology and Pathophysiology, Peking University School of Basic Medical Sciences, Beijing 100191, China; ^4^Beijing Key Laboratory of Bioactive Substances and Functional Foods, Beijing Union University, Beijing 100191, China

## Abstract

Epigenetic modifications like DNA methylation and histone acetylation play an important role in a wide range of brain disorders. Histone deacetylases (HDACs) regulate the homeostasis of histone acetylation. Histone deacetylase inhibitors, which initially were used as anticancer drugs, are recently suggested to act as neuroprotectors by enhancing synaptic plasticity and learning and memory in a wide range of neurodegenerative and psychiatric disorders, such as Alzheimer's disease (AD) and Parkinson's disease (PD). To reveal the physiological roles of HDACs may provide us with a new perspective to understand the mechanism of AD and to develop selective HDAC inhibitors. This paper focuses on the recent research progresses of HDAC proteins and their inhibitors on the roles of the treatment for AD.

## 1. Introduction

Alzheimer's disease (AD), including familiar AD and sporadic AD, is a progressive neurodegenerative disease and the most common form of dementia. This late-onset disorder is characterized by memory loss and cognitive impairment. The pathological features are neurofibrillary tangles (NFTs), insoluble *β*-amyloid (A*β*) plaques, and neuron loss [1]. According to the most convincing hypothesis at present, the “amyloid cascade hypothesis,” it is believed that the accumulated A*β* peptide leads to a complex cascade of neuronal apoptosis and results in the pathogenesis of AD [2, 3]. Increasing evidence supports the notion that some genetic changes in familiar AD, such as amyloid *β* precursor protein (APP), presenile 1, 2 (PS1&2), and apolipoprotein E, are linked to the overproduction of A*β* [4]. Although we have known a lot about both familiar AD and sporadic AD, it is still a long way to fully understand the pathogenesis of the diseases, especially sporadic AD. Epigenetics is a branch of genetics. Epigenetics studies the gene expression when the nucleotide sequences of a gene do not change, but rather other modification factors including histone acetylation and DNA methylation [5]. The epigenetic regulation offers a new way to understand AD, especially sporadic AD. DNA methylation has been previously proved to play a role in AD, and recently several studies suggested that histone acetylation is involved in the etiology of AD [6, 7]. Histone acetylation and deacetylation are catalytic by histone acetyltransferases (HATs) and histone deacetylases (HDACs), respectively. The level of histone acetylation plays an important role in regulating the chromatin condensation and gene transcription [8]. HDACs regulate the level of histone acetylation and further affect some downstream gene expression. Abnormal acetylation of histone is involved in the pathology of AD. HDAC proteins may be therapeutical targets to treatment for AD. HDAC inhibitors have been reported to improve the memory and cognition in the mouse model of AD. HDAC inhibitors may be alternative drugs to potentially protect the impairment of cognition in AD patients. However, HDAC proteins serve a very distinct function in the brain. Therefore, the use of pan-HDAC inhibitors (nonselective HDAC inhibitors) in the treatment of neuropsychiatric disorders should be careful. To identify which numbers of HDAC family are involved in memory and learning is helpful in discovering the pathological mechanism of AD and in developing selective HDAC inhibitors.

## 2. Histone Deacetylase and Histone Deacetylase Inhibition

### 2.1. Histone Deacetylase

HDAC proteins belong to an ancient protein family in many species. In fact, contrary to what is suggested by their names, certain HDACs mainly interact with nonhistone protein. In mammals, there are eighteen HDAC enzymes (Table 1), which are divided into four classes based on their homology to yeast: class I, class II, and class IV. Among these classes, class I, class II, and class IV are zinc-dependent enzymes, whereas class III is dependent on nicotinamide adenine dinucleotide (NAD^+^) [9, 10]. 

Class I of HDACs, which consists of HDAC 1, 2, 3, and 8, primarily localizes in the nucleus where they regulate histone acetylation. Class II of HDACs is divided into two subtypes: class IIa and IIb. Class IIa includes HDAC 4, 5, 7, and 9 and class IIb includes HDAC 6 and 10. Class IIa of HDACs shuttles between the nucleus and the cytoplasm in response to certain cellular signals, whereas class IIb of HDACs mainly locates in cytoplasm. In general, class III of HDAC is referred to as “sirtuins.” Class III of HDACs includes SirT1-7, which share homologous sequence with the yeast Sir2 protein; SIRT 3, 4, and 5 are mitochondrial proteins [11]. Class IV HDAC is known to have only one member, HDAC11, which contains a catalytic domain located at the N-terminus. HDAC11 seems to be closely related to HDAC3 and HDAC8 [12], and it also mainly locates in the cellular nucleus [13].

### 2.2. Histone Deacetylase Inhibition and Alzheimer's Disease

In recent years, a variety of HDAC inhibitors have been developed (Table 1). The pan-HDAC inhibitors widely used in clinical research are valproic acid, trichostatin A, sodium 4-phenylbutyrate, and vorinostat; these inhibitors interact with zinc-dependent HDAC protein (class I, class II, and class IV). Nicotinamide, as the precursor of NAD^+^, inhibits class III HDAC proteins [14]. 

Valproic acid (VPA), as well as lithium, inhibits A*β* peptide production in HEK293 cell transfected with Swedish APP_751_ [15]. VPA also significantly reduces A*β* plaque in AD transgenic mice. VPA decreases A*β* production by inhibiting GSK-3*β*-mediated *γ*-secretase cleavage of APP and alleviates the memory deficits in AD mouse model [16]. Ricobaraza et al. suggested that 4-phenylbutyrate (PBA) decreases the phosphorylation of tau based on an increase of an inactive form of the GSK-3*β* in Tg2576 mouse model of AD [17]. PBA reinstates memory in both young and old Tg2576 mice and reverses learning deficits through clearance of intraneuronal A*β* accumulation and mitigation of endoplasmic reticulum (ER) stress [18]. Nicotinamide, a competitive inhibitor of class III NAD^+^-dependent HDACs, restores cognitive deficits in 3xTg-AD mice. Nicotinamide selectively reduces phosphorylation of tau at Thr231 site and increases the acetylated *α*-tubulin [14]. Ding et al. found that tubacin-treated HEK cells transfected with tau significantly attenuates tau phosphorylation at T231 [19]. However, the untoward effects of pan-HDAC inhibitors limit their application to clinic [20, 21]. Tubacin and suramin are the most studied selective HDAC inhibitors; tubacin shows high selectivity for HDAC6 [22], and suramin inhibits the activity of NAD^+^-dependent class III SirT1 and SirT2 [23]. Here are the isoforms of HDACs, and the mainly used HDAC inhibitors are listed in Table 1. 

Above facts indicate that some HDAC proteins may have a close relationship with those key proteins which are involved in AD. HDAC inhibitors may be used to treat AD by regulating the activity of HDAC proteins and phosphorylation of Tau. However, the mechanism of HDAC-regulated cellular signaling in AD pathology is needed to further investigate. 

## 3. Acetylation of Histone and Alzheimer's Disease

Histone acetylation is involved in the gene expression through chromatin modification, and the acetylation was mainly in the N-terminal of histone H3 and H4. The cognitive deficit is one of the major clinical characteristics of AD patients. In mice, deregulation of histone acetylation is associated with age-dependent memory impairment. Aged mice display a specific deregulation of histone H4 lysine12 (H4K12) acetylation and fail to initiate a hippocampal gene expression which is involved in memory consolidation, and vorinostat-treated mice display significant increase of H4K12 acetylation and restore learning-induced gene expression [24]. More and more evidence suggests that dysregulation of histone H4 acetylation is involved in AD pathology. Kilgore et al. reported that there is no difference in H3 or H4 acetylation in the hippocampus area between 6-month-old APP/PS1 mice and wild-type mice, but a significant reduction was observed in H4 acetylation in old APP/PS1 mice. The class I HDAC inhibitors sodium valproate, sodium butyrate, or vorinostat elevate histone H4 acetylation and restore contextual memory in a mouse model of AD [25]. Similarly, Francis et al. found that there is no difference in the acetylation level of histone H4 between the 4-month-old wild-type and the APP/PS1 mouse. After contextual fear-conditioning training, the APP/PS1 mice display a reduced endogenous level of histone H4 acetylation, and the mice treated with trichostatin A (TSA), a class I and II HDAC inhibitor, had improved acetylated histone H4 level and were displayed hippocampal CA3-CA1 long-term potentiation (LTP) [26]. Ricobaraza et al. reported that the acetylation of histone H4 is decreased in the 16-month-old Tg2576 mouse brain compared with nontransgenic controls, while there is no difference in H3 acetylation. Phenylbutyrate, a class IIa HDAC inhibitor, ameliorates the cognitive deficits in Tg2576 mice and increases the neuronal acetylation of H4 and the expression of synaptic plasticity markers including GluR1, PSD95, and MAP2 [17]. What is more, in Tg2576 mouse model of AD, the phenylbutyrate restores the dendritic spine density of hippocampal CA1 pyramidal neurons and significantly increases the expression of plasticity-related proteins like the NMDA receptor subunit NR2B and the synaptic scaffold SAP102 [18]. Taken together, histone acetylation H4 is involved in the pathology of AD, and HDAC inhibitors may alter some important gene expression through regulating the histone acetylation.

## 4. Histone Deacetylases and Alzheimer's Disease

HDACs influence the level of histone acetylation, and HDAC inhibitors upregulate histone acetylation level and improve memory and learning. The HDAC inhibitors affect the activities of the proteins that play an important role in AD, like A*β*, GSK-3*β*, and tau protein. GSK-3*β*, as a main tau phosphokinase, is linked to several mechanisms involved in AD. Increasing evidence suggests that A*β* induces hyperphosphorylation of tau though the activation of GSK-3*β* [27, 28]. Therefore, inhibition of A*β*-induced deficits of histone acetylation and hyperphosphorylation of tau is helpful to treat AD. HDAC inhibitors downregulate HDAC activity and thus increase the acetylation level of histone. At present, however, most available HDAC inhibitors are nonselective for HDACs because it is not fully understood which subtypes of HDACs have effects on development of AD. Identifying which subtypes of HDAC family members are involved in the pathology of AD is needed to further investigate, specifically, impairment of memory and learning.

### 4.1. Class I HDAC

Mice overexpressing HDAC2, but not HDAC1, result in decreased synaptic plasticity, synapse number and memory formation, and vorinostat could rescue the synaptic number and learning impairments in HDAC2-overexpressing mice. Generally, HDAC2 negatively regulates learning and memory [35]. Akhtar et al. demonstrated that in mature neurons the upregulated level of HDAC2 affects the basic excitatory neurotransmission, implying that HDAC2 may play a role in synaptic plasticity [36]. 

McQuown et al. found that HDAC3-Flox-modified mice (deletion of HDAC3 in the hippocampus of CA1 area) or the mice treated with RGFP136 (selective inhibitor of HDAC3) increase histone acetylation and significantly enhance long-term memory, and the expression of the genes of nuclear receptor subfamily 4, group A, member 2 (Nr4a2) and c-Fos is implicated in long-term memory [37]. Besides, Bardai and d'Mello suggested that HDAC3 is a protein with strong neurotoxic activity, and the toxic effect is cellular selective. HDAC3 is directly phosphorylated by GSK-3*β*, and inhibition of GSK-3*β* protects against HDAC3-induced neurotoxicity [38].

### 4.2. Class II HDAC

HDAC6, as a cytosolic enzyme, catalyzes several nonhistone proteins, such as tubulin and HSP90 deacetylase [39, 40]. HDAC6 protein level in AD brains is significantly increased in cortex and hippocampus compared with the normal brains. Tubacin (a selective inhibitor of HDAC6) attenuates site-specific phosphorylation of tau, suggesting that HDAC6 plays a role in the AD [19]. The selective inhibition of HDAC6 dramatically enhances mitochondrial movement in hippocampal neurons. GSK-3*β* may be involved in the active regulation of HDAC6 by phosphorylation pathway. Therefore, abnormal mitochondrial transportation could be resulted from the misregulation of HDAC6 by GSK-3*β* [41]. Besides, oxidative stress is the main pathological feature of AD, and selective inhibition of HDAC6 protects against oxidative-stress-induced neurodegeneration and promotes neurite outgrowth in cortical neurons [42]. 

HDAC4 is mainly localized in the cytoplasm of brain tissue, and abnormal expression of nuclear-localized HDAC4 promotes neuronal apoptosis while inactivation of HDAC4 suppresses neuronal cell death [43]. Therefore, HDAC4 may play an important role in nerve function.

### 4.3. Class III HDAC

Gao et al. found a significant reduction of SirT1 in the parietal cortex of AD patients compared with the control, and the accumulation of A*β* and tau in the AD patients may be associated with the loss of SirT1 [29]. Julien et al. suggested that the mutant mice with the lack of SirT1 show impairment in memory and synaptic plasticity, and SirT1 modulates synaptic plasticity and memory formation partly via the way of upregulation of miR-134 [30]. Therefore, SirT1 could be a target for the treatment of neurodegenerative disorders. Additionally, overexpression of the NAD^+^-dependent deacetylase SirT1 in a mouse model of AD reduces the production of A*β* and formation of plaques through activating the transcription of gene encoding the *α*-secretase ADAM10. SirT1 also regulates the Notch pathway, which repairs neuronal damage in brains [31]. 

The level of *α*-tubulin acetylation is known to play an important role in microtubule stability and SirT2 decreases the level of *α*-tubulin deacetylation [14]. Taylor et al. suggested that inhibition activity of SirT2 deacetylase reduces total cholesterol in primary striatal neurons [32]. The above facts hint that Sir2 is involved in the pathology of AD.

Kawamura et al. found out that RNAi-mediated SirT3 knockdown increases mitochondrial reactive oxygen species (ROS) generation in mouse fertilized eggs, and the mitochondrial ROS generation is accompanied by the p53 upregulation in SirT3-knockdown mouse embryos [33]. Additionally, the primary cultured mouse cortical neurons treated with NMDA induce massive ROS production as well as the increase of mitochondrial SirT3, while overexpression of SirT3 significantly reduces the mitochondrial ROS generation [34]. Therefore, the SirT3 appears to play a role in the protection of the nervous system against excitotoxicity.

## 5. Summaries

**Figure 1:**
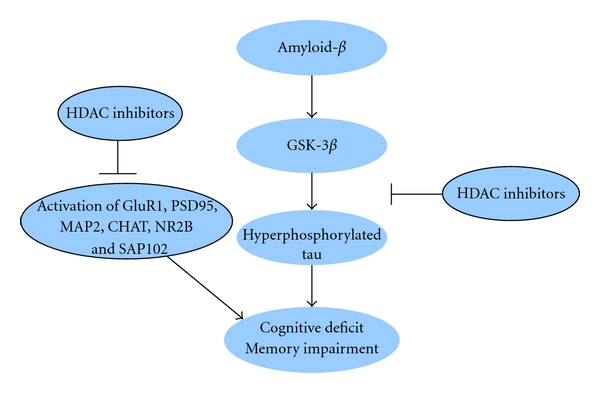
The role of HDAC inhibitors in Alzheimer's disease. First, HDAC inhibitors inhibit A*β*-induced hyperphosphorylation of tau protein. Second, HDAC inhibitors alter the expression of important genes which participate in the learning and memory.

AD is one of the most common forms of dementia. Currently, the pathology of this disease is not fully understood. “Amyloid cascade hypothesis” states that the increased A*β* causes the disease progress, such as cognitive deficits, memory impairment, and neuron loss. Accumulating evidence supports the view that the HDAC proteins may be involved in its development. The HDAC proteins may regulate the level of histone acetylation and then alter the expression of some important genes which are involved in the memory and cognition and pathology of AD. HDAC inhibitors may ameliorate cognitive deficits and memory impairment in AD animal models. The potential pathways of HDAC inhibitors reversing A*β*-induced neurotoxicity may lie in that (1) HDAC inhibitors inhibit A*β*-induced hyperphosphorylation of tau protein; (2) HDAC inhibitors may regulate the expression of important genes that participate in the learning and memory (Figure 1). However, many issues need to be resolved before these inhibitors can be used to treat AD. For example, it is not yet clear which subtypes of HDACs are associated with the AD and which selective HDAC inhibitors would be effective to treat AD. Further researches are needed to clarify the exact role of HDAC proteins and to develop their selective inhibitors in the pathology of AD. 

## Figures and Tables

**Table 1:** HDAC isoforms and main used of pan-HDAC inhibitors.

Histone subtype	Protein	Localization	Main HDAC inhibitors
Class I (Zn++-dependent)	HDAC1, 2, 3, and 8	Mainly nucleus	Valproic acid, butyrate, vorinostat, trichostatin A, RGFP136 (HDAC3)
Class IIa (Zn++-dependent)	HDAC4, 5, 7, and 9	Nucleus/cytoplasm	Trichostatin A, phenylbutyrate
Class IIb (Zn++-dependent)	HDAC6 and 10	Mainly cytoplasm	Tubacin (HDAC6), trichostatin A
ClassIII (NAD+-dependent)	SirT1, 2, 3, 4, 5, 6, and 7	Nucleus/cytoplasm/Mitochondria	Nicotinamide, suramin (SirT1 and SirT2),
Class IV (Zn++-dependent)	HDAC11	Mainly nucleus	
